# 

**Published:** 1895-07

**Authors:** 


### TABLE OF CONTENTS.

ORIGINAL COMMUNICATIONS. PAGE

The History of the Cusps of the Human Molar Teeth. By Henry Fairfield Osborn . . . . . 389

The Application of some Principles of Law to the Practice of Dentistry. By W. A. Purrington, Esq., New York . . . . . 403

Theory and Results. By G. Lenox Curtis, M.D., D.D.S., New York City . . . . . 419

REPORTS OF SOCIETY MEETINGS.

New York Odontological Society . . . . . 429

New York Institute of Stomatology . . . . . 440

EDITORIAL.

Questions and Answers . . . . . 453

The Annual Conventions . . . . . 455

Pennsylvania State Dental Society . . . . . 456

OBITUARY.

Dr. Arnold C. Hawes . . . . . 457

Dr. Thomas H. Musgrove . . . . . 458

Dr. Elisha G. Tucker . . . . . 459

CURRENT NEWS.

American Dental Association Meeting . . . . . 460

Pennsylvania State Dental Society . . . . . 461

American Dental Society of Europe . . . . . 462

National Association of Dental Faculties . . . . . 462

To arrange for Interstate Meeting, Missouri . . . . . 462

